# Involvement of Rho GAP GRAF1 in maintenance of epithelial phenotype

**DOI:** 10.1080/19336918.2016.1227910

**Published:** 2016-09-02

**Authors:** Miriam Regev, Helena Sabanay, Elena Kartvelishvily, Zvi Kam, Alexander D. Bershadsky

**Affiliations:** a Department of Molecular Cell Biology, Weizmann Institute of Science, Rehovot, Israel; b The Chaim Sheba Medical Center, Tel-Hashomer Hospital, Israel; c Mechanobiology Institute, National University of Singapore, Singapore

**Keywords:** anchorage dependence, cell-cell junctions, focal adhesions, cell migration, EMT, E-cadherin, MCF10A

## Abstract

Adhesion of epithelial cell to each other and to extracellular matrix, as well as cell migration ability and cytoskeleton organization undergo significant alterations in the course of neoplastic transformation, but regulatory mechanisms involved in these processes are not fully understood. Here, we studied the role of a Rho GAP protein GRAF1 (*G*TPase *R*egulator *A*ssociated with *F*ocal adhesion kinase-*1*) in the regulation of the epithelial phenotype in cells of breast derived, non-malignant, MCF10A cell line. GRAF1 was shown to be localized to cell-cell junctions, and its depletion resulted in accelerated cell migration velocity, elongation of the cells and cell colonies, impaired monolayer integrity and significant disruption of desmosomes with a loss of associated keratin filaments. These processes were accompanied by formation of larger focal adhesions, an increased number of contractile actin stress fibers, reduction in epithelial markers and increase in mesenchymal markers such as epithelial-mesenchymal transition (EMT)-specific transcription factors Snail-1 and Snail-2, as well as N-cadherin, and vimentin. Moreover, unlike control cells, GRAF1 knocked-down cells demonstrated anchorage-independent growth in soft agar. GRAF1 expression in several highly invasive breast cancer cell lines was low, as compared to the non-malignant MCF10A cells, while overexpressing of GRAF1 in the malignant BT-549 cell line led to a decrease of mesenchymal markers, especially the Snail-1 and 2. Altogether, our analysis suggests that GRAF1 plays a role in the maintenance of normal epithelial phenotype and its depletion leads to an EMT-like process that might be involved in neoplastic transformation.

## Introduction

Epithelial to mesenchymal transition (EMT)^1^ is a process shown to be fundamental to both normal development, and the progression of malignant epithelial tumors.^2,3^ In the course of EMT, epithelial cells lose cell–cell adhesion structures such as adherens junctions and desmosomes, rearrange their cytoskeleton, develop front-rear polarity and enhance migration.^4^ Activation of the transcriptional regulators, Snail-1(formerly Snail)^5^ and Snail-2 (also known as Slug), as well as several others, is thought to underlie the changes in gene expression patterns occurring during EMT.^6^ A hallmark of EMT is the loss of epithelial E-cadherin and the gain of mesenchymal N-cadherin expression. This cadherin switch^7^ leads to a drastic change in the adhesive properties of the cell; N-cadherin expression also promotes increased cell migration and invasion.^8,9^

The disassembly of junctional complexes and the changes in cytoskeletal organization that occur during EMT are orchestrated by alteration of activity of intracellular effector molecules, such as members of the small Rho GTPase family as well as Src-family protein tyrosine-kinases.^6^ Among RhoGTPases, the best-characterized molecules are RhoA, RhoB, RhoC, Rac1 and Cdc42 that regulate actin cytoskeleton. In particular, the active (GTP-bound) RhoA stimulates myosin II-driven contractility through activation of Rho kinase (ROCK), and promotes actin polymerization through activation of Diaphanous family formins.^10,11^ As a result, cells develop contractile myosin-II containing actin bundles (stress fibers) associated with a special type of integrin-mediated cell-matrix adhesions known as focal adhesions.^11^

RhoA and RhoC also play a role in neoplastic processes: their overexpression was detected in a large variety of human tumors.^12,13^ In particular, protein levels of RhoA were significantly higher in breast tumors, as compared to normal mammary tissue.^14^ Moreover, activation of RhoA was shown to promote breast cancer metastasis.^15^ Therefore, cellular mechanisms regulating activity of Rho proteins are important in the processes of tumor development and metastasizing.

Rho GTPase activation is tightly controlled by 3 groups of regulatory proteins, guanine nucleotide exchange factors (GEF), GTPase-activating proteins (GAP), and guanine nucleotide dissociation inhibitors (GDI). In this study, we focus on a RhoGTPase-activating protein GRAF1 (GTPase Regulator Associated with Focal adhesion kinase), which was shown to exert GAP activity toward RhoA and Cdc42 and binds to Focal Adhesion Kinase via its SH3 domain.^16^ GRAF1 (known also as ARHGAP26) is a member of the ARHGAP family of proteins. In addition to GAP and SH3 domains, it also contains a BAR-PH domain that underlies its involvement in clathrin-independent endocytosis.^17,18^ GRAF1 was reported to have 2 isoforms: the “A” isoform mostly expressed in leukocytes, and the “B” isoform, highly expressed in many types of epithelial tissues, in particular in the mammary glands and also in nervous tissues.^19^

Since GRAF1 is a physiological negative regulator of Rho activity, we decided to study whether this protein participates in the regulation of the epithelial phenotype and EMT processes. MCF10A cells were selected as an appropriate cell type for these studies because of their phenotypic plasticity. Single MCF10A cells display typical EMT-like changes, in contrast to an epithelial phenotype seen in MCF10A cells organized in groups, or growing as monolayers.^20^

In our study, we investigated the effect of GRAF1 knockdown on morphology, cytoskeletal organization, cell-cell and cell-matrix adhesion and motility of MCF10A cells. We found that GRAF1 depletion triggers the process of epithelial to mesenchymal transition in these cells. In addition, we found that loss of GRAF1 was typical for neoplastically transformed lines of breast cancer origin. Thus, GRAF1 function appears to be important for the maintenance of the normal phenotype in mammary gland epithelium.

## Results

### Cell shape, cell-cell junction integrity and cell migration velocity changes in MCF10A epithelial cells upon knockdown of RhoGAP GRAF1

**Figure 1.**
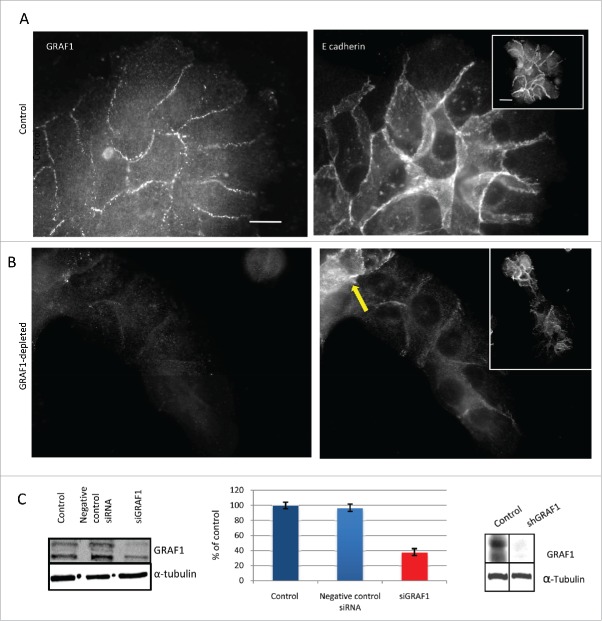
GRAF1 knockdown in MCF10A cells infected with lentivirus containing shRNA directed against GRAF1. (A) Localization of GRAF1 (left) and of E-cadherin (right) in control cells. (B) GRAF1 shRNA-expressing MCF10A cells (left) visualized by staining with GRAF1 antibodies. Note the presence of GRAF1 in control cells and its significant decrease upon shRNA expression. The level of junctional E-cadherin is high in control cells (A, right), but decreased upon GRAF1 depletion (B, right). Note some cells demonstrating high E-cadherin intensity (arrow). Scale bar: 5 μm. In insets: Islands of control (A, right) and GRAF1-depleted cells (B, right). The cell-cell junctions are visualized by E-cadherin-antibody. Notice that morphology of epithelial islands which became elongated upon GRAF1 depletion. Scale bar: 5 μm. (C) Left: Validation of siRNA-mediated GRAF1 knockdown. Western blot shows significant decrease in expression of heavier GRAF1 splicing B isoform and some decrease of A isoform. Middle: Quantification of expression of the GRAF1 protein (heavier isoform) by RT-PCR; Right: Validation of GRAF1 knockdown in the stable shGRAF1 expressing line.

Here, we tested the effect of GRAF1 silencing on the phenotype of cultured non-transformed MCF10A human mammary epithelial cells. Transient GRAF1 depletion was obtained using a siRNA SMARTpool for GRAF1 (isoform B), while a stable line of GRAF1 isoform B-depleted MCF10A cells was produced by lentiviral infection with the plasmid encoding the corresponding shRNA, and subsequent selection. Decrease of GRAF1 expression obtained by siGRAF1 was assessed on western blot and real time PCR. On western blot, an 80% decrease in the GRAF1 protein encoded by isoform B was seen (Fig. 1C, left). We obtained also some reduction in the GRAF1 isoform A expression. On real-time PCR, the decrease of GRAF1 RNA expression was up to 40% (Fig. 1C, middle). We obtained an almost complete decrease of both isoforms of GRAF1 in the stable line expressing shGRAF1 (Fig. 1C, right). Immunofluorescence staining of control and GRAF1-depleted cells with α-tubulin antibody revealed no differences between the 2 groups (Supplementary Fig. 1A).

**Figure 2.**
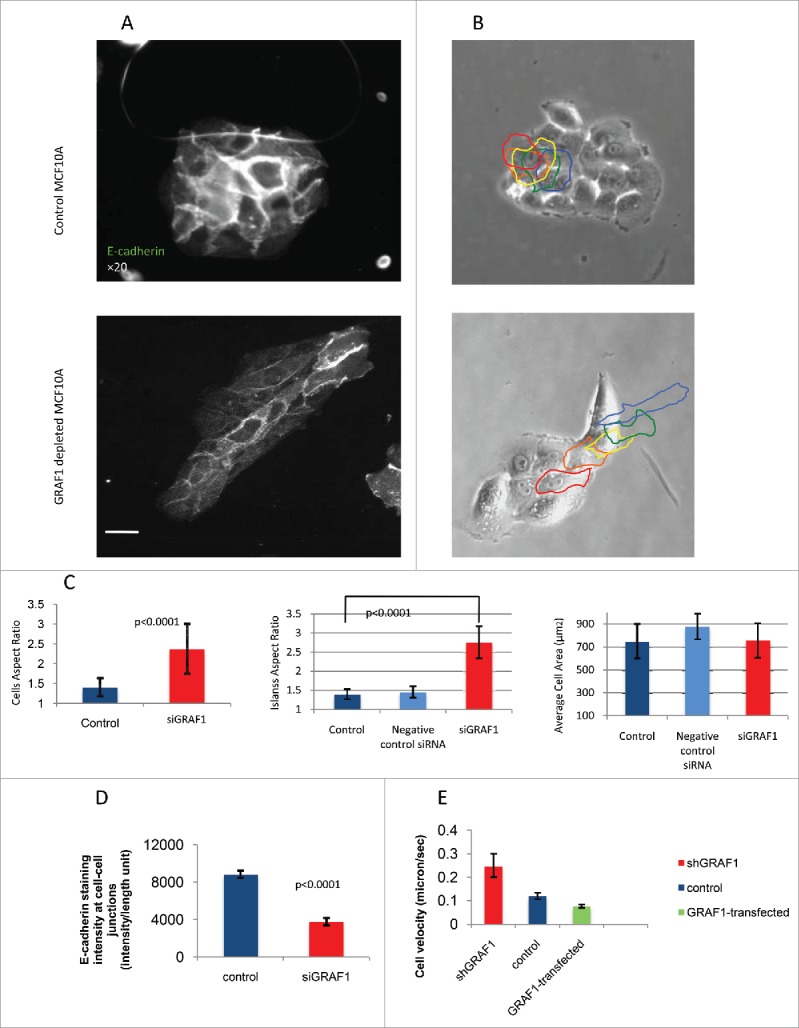
Morphology and motility of GRAF1-depleted cells. (A) E-cadherin antibody staining reveals that a control colony of MCF10A cells (top) displays, on average, higher levels of E-cadherin at cell-cell junctions, than a colony from a GRAF1-depleted MCF10A cell culture (bottom). Notice, however, that some cells in the GRAF1-knockdown colony demonstrate relatively high E-cadherin levels. Scale bar: 10 μm. (B) Live-cell imaging of control (top) and GRAF1-depleted MCF10A cells (bottom). Sequential position of a single cell in the island is marked by borders colored in spectrum order (red = 0 time, orange = 4 hours, yellow = 8 hours, green = 12 hours, blue = 20 hours). Control cells are not elongated; rather, form approximately circular islands. Displacement of cells in the island during the 24-hour observation period did not exceed the cell diameter. GRAF1-depleted cells form an elongated island, and move from the interior of the island to the cell periphery. Scale bar: 10 μm. (C) Morphological characteristics of control and GRAF1-depleted cells and their colonies. Left: Cell aspect ratio. Middle: Colony aspect ratio. Right: Cell area. Notice that both cells and their colonies became elongated upon GRAF1 depletion, while the cell's projected area did not change. p values were calculated by a Student's t-test. Measurements were performed in at least 50 cells per sample. (D) Quantification of E-cadherin staining intensity at cell-cell contacts. Image analysis was performed using software designed in-house. Measurements were performed for 50 fields in each sample. Decrease of E-cadherin levels upon GRAF1 knockdown is highly statistically significant (p < 0.001). (E) Increase of migratory velocity in GRAF1-depleted cells, as compared to control cells and GRAF1-transfected cells. Velocities were measured by tracking the centers of the cell nuclei. Quantification included 100 cells for each sample and was undertaken using a specially designed tracking program (see “Materials and methods”).

In control MCF10A cells, GRAF1 localizes to the cell-cell junctions partially overlapping with E-cadherin (Fig. 1A). While control cells formed circular epithelial islands (Fig. 1A insight and 2A, top), depletion of GRAF1 led to formation of elongated islands (Fig. 1B insight and Fig. 2A bottom). Strikingly, GRAF1-depleted cells were characterized by a significant decrease in the E-cadherin junctional level (Fig. 1B and Fig. 2D). An off-target effect was ruled out by transfection of MCF10A cells with 4 individual siRNAs targeted to GRAF1, which comprised the GRAF1 siRNA SMARTpool. A significant reduction in E-cadherin and β-catenin at cell-cell junctions was obtained after cell transfection with 3 out of 4 individual SMARTpool siRNAs, as was demonstrated by immunofluorescence staining (Supplementary Fig. 1D-H) and by Western blot (Supplementary Fig. 1B,C).

It is worth noting that among cells depleted of GRAF1, some cells still demonstrated high E-cadherin intensity. These cells were usually gathered at one extremity of the cell group, usually at the rear (Fig. 1B, arrow). Measurements of intensity of E-cadherin-antibody staining at the cell-cell contact showed an average decrease in E-cadherin junctional level in GRAF1-depleted cells to less than 40%, relative to that in control cells (Fig. 2D).

A series of assays, was performed to characterize changes in cell shape and motility upon GRAF1 knockdown. Cells lacking GRAF1 displayed a fourfold-higher aspect ratio when compared to controls (Fig. 2C, left), while the average cell area remained unchanged (Fig. 2C, right). Aspect ratio assessment confirmed the visual impression that islands formed by GRAF1-depleted cells are significantly more elongated than the typical epithelial islands formed by control cells or “scrambled” siRNA- transfected cells (Fig. 2C, middle). Thus, the degree of spreading of the GRAF1-knockdown cells remains unchanged, but the cells become more elongated, and form elongated groups.

Morphology of GRAF1-depleted cells was further assessed in live cells, by means of phase contrast-microscopy. In the elongated groups of GRAF1-depleted cells, one or 2 cells extended large lamellas at the epithelial island's leading edge, while others appeared to be non-protrusive. Moreover, the cells from the interior often migrated through the entire island toward the leading edge, disrupting the cell-cell junctions. As a result, some epithelial islands underwent scattering and disintegration during the 20-hour period of observation. Control epithelial islands, on the other hand, moved as a whole, preserving mutual contacts with each other, and the circular shape of the epithelial island (Fig. 2B; Videos 1 and 2).

Migration velocity of MCF10A control cells, GRAF1-depleted cells, and GFP-GRAF1 overexpressing cells was measured by tracking the centers of the nuclei for 20 hours, with a 20-minute time lapse between frames. In all cases, the cells that were located at the edges of a group, rather than the cells embedded in a monolayer, were tracked. GRAF1-depleted cells displayed a twofold-higher migration velocity as compared to controls, while GRAF1-overexpressing cells moved more slowly than controls (Fig. 2E).

**Figure 3.**
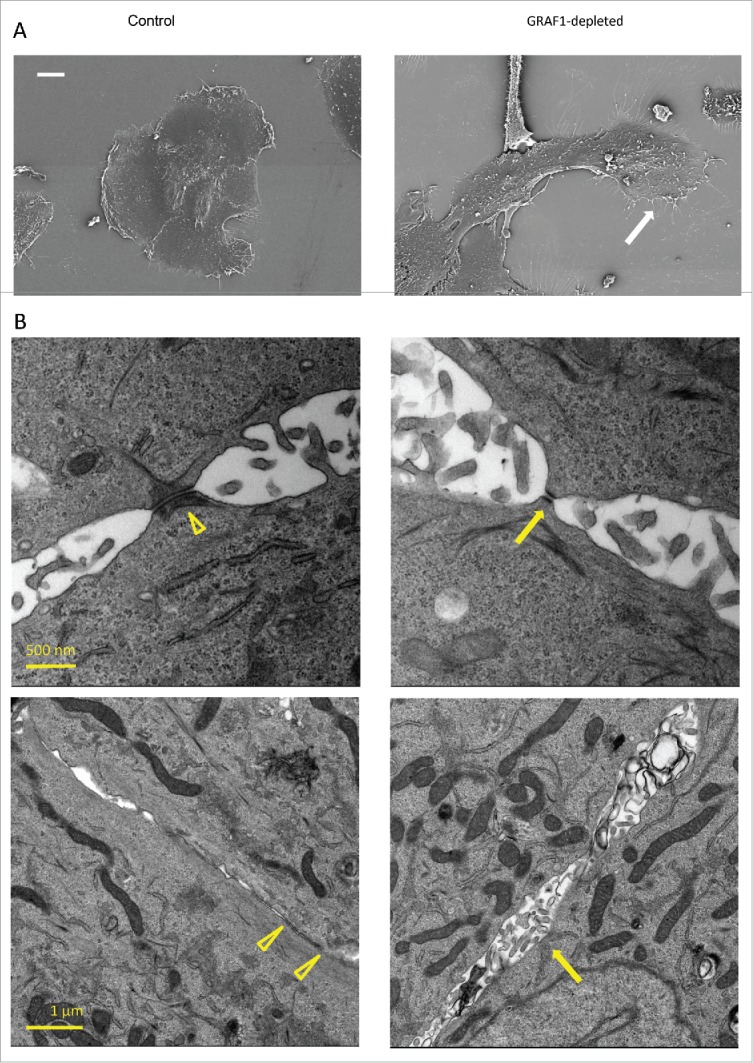
Electron microscopy analysis of alterations in cell morphology and cell-cell junction organization upon GRAF1 knockdown. (A) Scanning electron microscopy images of the small colonies of control and GRAF1-depleted cells. Note that the colony of GRAF1 depleted cells, as well as individual cells, are more elongated. The “leading” cell (denoted by a white arrow) of the GRAF1 depleted colony displays a large lamellum and numerous filopodia. (B) Disruption of cell-cell contacts seen by transmission electron microscopy. Upper row: Note smaller desmososmes and less prominent arrays of intermediate filaments (presumable cytokeratin filaments) in GRAF1-depleted cells (yellow arrow in the right image), as compared to controls(arrowhead in the left image). Lower row: The control cells (left) are characterized by wide adherens junctions (arrowheads), while GRAF1-depleted cells (right) demonstrate instead open intercellular spaces with multiple microvilli (yellow arrow).

Scanning and transmission electron microscopy (SEM and TEM) were further used to investigate the structural alterations induced by GRAF1 knockdown in MCF10A cells. As noted in the phase-contrast images, GRAF1-depleted cells demonstrated an elongated cell shape and protruding lamellipodia at the island's leading edges (Fig. 3A, right, white arrow). In addition, these cells displayed more numerous microvilli distributed over the entire cell surface, as well as structures resembling filopodia emerging from the cell edges, than control MCF10A cells (Fig. 3A).

TEM was used to assess the integrity of cell-cell junctions in control and GRAF1-depleted cells. In agreement with previously published results,^21^ control MCF10A cells demonstrated typical adherens junctions and desmosomes, but very few tight junctions. In GRAF1-depleted cells, the gaps between cells in the area of the adherens junctions were broader, with a larger intercellular space (Fig. 3B, lower panel, yellow arrow) than that seen in controls (yellow arrowheads), and multiple microvilli were seen protruding into this space. The number of desmosomes was apparently fewer than in control cells, even though quite a few desmosomes were still seen upon GRAF1 knockdown. These desmosomes, however, were smaller (Fig. 3B, upper panel, yellow arrow) and associated with less prominent arrays of cytokeratin fibers, as compared to the desmosomes in control cells (arrowhead). Thus, GRAF1 depletion induced severe changes in the integrity of cell-cell junction complexes.

### Changes in the actin cytoskeleton and focal adhesions in MCF10A after GRAF1 depletion

**Figure 4.**
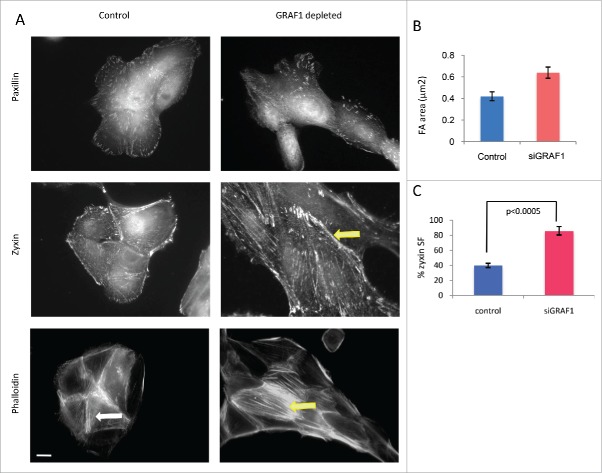
Junctional and cytoskeletal changes following GRAF-1 depletion. (A) Upper row: GRAF1-depleted cells display larger focal adhesions (visualized by staining with paxillin antibody), as compared to control cells. Middle row: Zyxin is localized to focal adhesions and stress fibers. In GRAF1-depleted cells, stress fiber localization of zyxin is more prominent than in control cells (arrow). Lower row: Actin stress fibers (visualized by phalloidin staining and indicated by arrows) in GRAF1-depleted cells are more robust and form parallel arrays (yellow arrow), not observed in controls (white arrow).Scale bar: 5 μm. (B) Measurement of focal adhesion size in control and GRAF1- depleted cells. At least 50 cells from each sample were measured. Average size of focal adhesions in GRAF1-depleted cells is 1.5 larger than that of controls. (C) Quantification of zyxin-positive stress fibers in control and GRAF1-depleted cells; not less than 100 cells in about 25–30 microscope fields from each sample were analyzed. Note the increase of zyxin-positive stress fibers in GRAF1- depleted cells, as compared to controls (p < 0.0005 according Student's t-test).

Focal adhesions (FAs) are dynamic molecular complexes connecting the extracellular matrix with the actin cytoskeleton of adherent cells, via integrin-family transmembrane receptors and a plethora of associated linking proteins.^22,23^ In essence, FAs are signaling structures, transmitting to cells information about the biochemical and physical characteristics of their microenvironment; they are also involved in the organization of the actin cytoskeleton, cell shape and migration, and matrix remodeling.^24^ To assess how focal adhesions are altered upon GRAF1 depletion, we used immunofluorescence staining to visualize 2 characteristic components of focal adhesions, paxillin and zyxin. Focal adhesions visualized by anti-paxillin antibody were 1.5-fold larger in GRAF1-depleted cells than in controls (Fig. 4A, upper panel, and 4B). A similar increase in focal adhesion size was also observed by visualization of focal adhesions with anti-zyxin antibodies (Fig. 4A, middle panel). Interestingly, visualization of zyxin revealed that this protein localized not only to focal adhesions, but also to the actin bundles (“stress fibers”) associated with them (Fig. 4A, middle panel, arrow and Fig. 4C). The percentage of cell, in which zyxin was localized to stress fibers was significantly higher in GFAF1-depleted cells than in control ones (p < 0.0005 according to t-test). In addition, in cells lacking GRAF-1, actin stress fibers were more prominent than in control MCF10A cells (Fig. 4A, lower panel). While in control cells, actin staining with phalloidin revealed mainly radial-oriented bundles (Fig. 4A, lower panel, white arrow), in GRAF1 knockdown cells, numerous parallel stress fibers were usually oriented along the long axes of the individual cells, parallel to the long axis of the entire island (Fig. 4A, lower panel, yellow arrow).

### GRAF1 depletion in MCF10A cells is accompanied by an increase in mesenchymal markers

The fact that GRAF1 depletion led to increased cell elongation, reduction of E-cadherin at the cell-cell junctions, and an increase in the cell's migratory ability, raised the question of whether depletion of GRAF1 could induce changes that occur in the course of EMT processes.^6,25^

**Figure 5.**
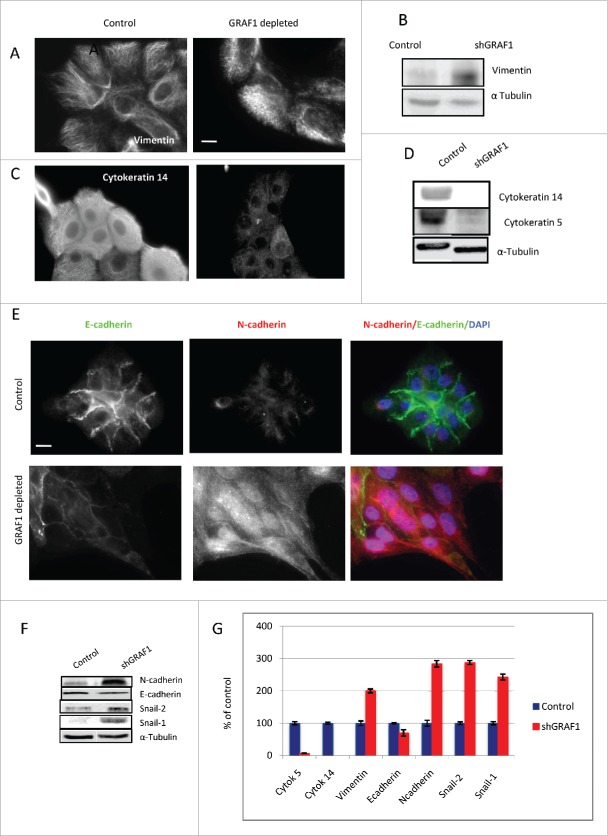
Markers of EMT in control and GRAF1-depleted MCF10A cells. (A) Increase in vimentin filament density, as visualized by immunofluorescence staining of control and GRAF1- depleted cells with anti-vimentin antibody. Scale bar: 5 μm. (B) Western blot with anti-vimentin antibody reveals an increase in vimentin expression in GRAF1-depleted versus control cells. (C) Loss of cytokeratin 14, following GRAF1 depletion. (D) A major decrease in cytokeratin 14 and 5 in GRAF1-depleted cells, indicated by Western blot. (E) Replacement of E-cadherin by N-cadherin upon GRAF1 depletion. Left column: E-cadherin contents at cell-cell junctions is significantly higher in control cells than in GRAF1-depleted cells. Middle column: N-cadherin contents in the same pair of colonies as seen in the upper panel. Note the significant increase in both cytoplasmic and junctional N-cadherin level in GRAF1-depleted cells, as compared to controls. Right column: Merged images of E-(green) and N-(red) cadherin in control and GRAF1-depleted cells. Nuclei are stained by DAPI (blue). Scale bar: 5 μm. (F) Western blot analysis of E- and N-cadherin contents, as well as markers of EMT Snail-2 /Snail-1 in control and GRAF1- depleted MCF10A cells. An increase in N-cadherin and EMT-characteristic transcriptional factors Snail-2 and Snail-1 is seen. Alpha-tubulin levels did not change upon GRAF1 depletion. (G) Quantification of the levels of expression of EMT markers in GRAF1-depleted cells is shown as percents of control. The α-tubulin level was used as a loading control; values representing the expression levels of all proteins of interest were normalized to the α-tubulin level.

Cytokeratins 5 and 14, classic epithelial markers, decreased dramatically in GRAF1-depleted cells, as demonstrated by both immunofluorescence staining (Fig. 5C) and Western blotting (Fig. 5D). At the same time, the amount of mesenchimal marker vimentin,^26^ as well as density of vimentin-positive intermediate filaments, increased upon GRAF1 depletion (Fig. 5A and B). Alpha-tubulin was used as a loading control in these experiments.

Replacement of epithelial E-cadherin for mesenchymal N-cadherin is known to be a hallmark of EMT. Indeed, in our study, N-cadherin partially replaced E-cadherin in GRAF1-depleted cells (Fig. 5E); however, the replacement of E-cadherin by N-cadherin occurred in a non-uniform fashion. We noted that the replacement of E-cadherin by N-cadherin was more prominent at the leading edges of elongated cell groups (corresponding to cells extending protrusions in the direction of collective movement) while at the opposite end of the same group (rear), E-cadherin was still strongly expressed [Fig. 1B, arrow and Fig. 5E (lower panel)], and expression levels of N-cadherin were lower. Interestingly, GRAF1 knockdown brought about a significant increase, not only in junctional but also in total (cytoplasmic) N-cadherin (Fig. 5E-G), while a decrease in junctional E-cadherin was not accompanied by an equally strong decrease in its cytoplasmic levels (compare Figs. 2D, 5G).

Augmented levels of the transcription factors snail-1 and snail-2 constitute a well-established mechanism of EMT, underlying the alterations in expression of many downstream target genes.^27^ We found that in GRAF1-depleted cells, the expression of snail-1 and snail-2 increased dramatically (Fig. 5G). The expression of snail-1 was barely detectable in control cells, but very prominent in the cells lacking GRAF1, the expression of snail-2 showed a 2-fold increase. Altogether, the alterations in expression of the EMT markers upon GRAF1 knockdown are summarized in Fig. 5G. In view of these data, we conclude that GRAF1-depleted cells demonstrate an EMT-like phenotype.

### MCF10A GRAF1-depleted cells are able to form colonies in soft agar gel

“Anchorage independence” - that is the ability of cells to form colonies in a semi-solid medium in conditions of substrate deprivation – is a very typical feature of neoplastically transformed cells. It was previously shown that MCF10A cells that underwent an EMT process following ras transformation, acquire the ability to form such colonies.^28^ In our study, control and GRAF1-depleted MCF10A cells were cultured for 3 weeks in soft agar gel enriched by growth medium. The colonies formed during this period were imaged using an inverted microscope, and classified according to size (Table 1). While in control MCF10A cultures, only single cells or cell fragments having less than 20μm^2^ area were detected, GRAF1-depleted cells formed numerous cell aggregates with area between 20 and 100μm^2^, and some bigger colonies having 100-200 μm^2^ area (Table 1). Thus, we can conclude that GRAF1 knockdown made MCF10A cells less anchorage-dependent, a finding that supports the hypothesis that GRAF1 knockdown in MCF10A cells promotes EMT.

**Table 1.** Number of soft agar colonies per field. Number of colonies was counted using x10 objective of Zeiss, Axioplan inverted microscope. Data of one typical experiment are presented.

Number of colonies per field in GRAF1-depleted cells	Number of colonies per field in control cells	Colonies area
10.9 ± 2.33	5 ± 5.9	Small (<20 µm^2^)
17.7 ± 4.67	0	Medium (20–100 µm^2^)
1.7 ± 0.67	0	Large (100–200 µm^2^)

### Correlation of GRAF1 low expression with invasiveness and EMT phenotype in breast cancer-derived cell lines

**Figure 6.**
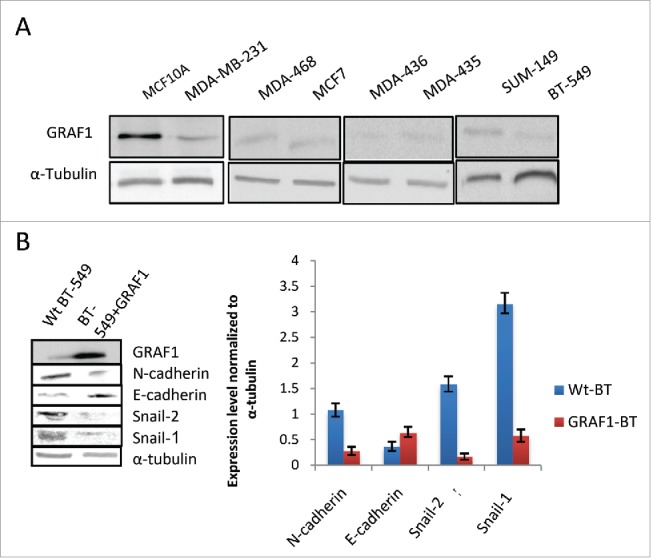
Effect of GRAF1 expression on the phenotype of breast cancer-derived cell lines. (A) Seven different breast cancer lines demonstrate lower level of GRAF1 expression, as compared to non-malignant MCF10A cells. Alpha-tubulin is used as a loading control. The characteristics of the cell lines are described in Supplementary Table 1. (B) Levels of expression of EMT markers in neoplastically transformed BT-549 cells, before and after GRAF1 overexpression in these cells. Left: Western blot. Right: Quantification of protein expression levels, normalized to α-tubulin levels in corresponding cells.

The GRAF1 gene was reported to be disrupted in juvenile myelomonocytic leukemia^29^ and aberrant methylation of the GRAF1 promoter was detected in patients with acute myeloid leukemia.^30^ To reveal the possible correlation between GRAF1 levels and cancer cell phenotype, we assessed GRAF1 expression levels in 7 different cell lines, all of breast cancer origin: invasive MDA-MB-231, MDA-MB-468, MDA-MB-435, and SUM-149, which preserved an epithelial-like morphology, MDA-MB-436 and BT-549, characterized by a polymorphic morphology, and the non-invasive neoplastic MCF7 line (Supplementary Table 1). Unlike non-malignant MCF10A cells, all 7 selected cell lines displayed low expression levels of GRAF1 (Fig. 6A), suggesting that the GRAF1 gene is involved in maintenance of the normal epithelial phenotype, and may therefore be considered as a tumor suppressor-type gene.

In order to determine whether overexpression of GRAF1 in neoplastic cells would reverse their phenotype, we selected the BT-549 cell line, characterized by a polymorphic mesenchymal morphology, and expressed GFP-GRAF1 in these cells. Roughly 72 hours after transfection, GFP-GRAF1 expressing BT-549 cells displayed a decrease in snail-1 and snail-2 expression to 20% and 10%, of their levels in non-transfected cells, respectively (Fig. 6B). Regarding the N-to E-cadherin switch, a 45% elevation in E-cadherin levels, and a decrease of N-cadherin to 27% of its level in wild-type BT-549 cells, were observed (Fig. 6B). These data suggest that GRAF1 expression led to a partial reversal of the mesenchymal to epithelial phenotype in neoplastic cells.

### Involvement of Rho family GTPases in the GRAF1 knockdown-induced EMT-like phenotype

Since the major biological function of GRAF1 known thus far is the modulation of Rho activity we examined whether GRAF1 knockdown affects the activity of RhoA in MCF10A GRAF1-depleted cells. We also studied the activity of Rac1, another major Rho family protein, which is known to be involved in regulating the formation of cell lamellipodia.^31^

**Figure 7.**
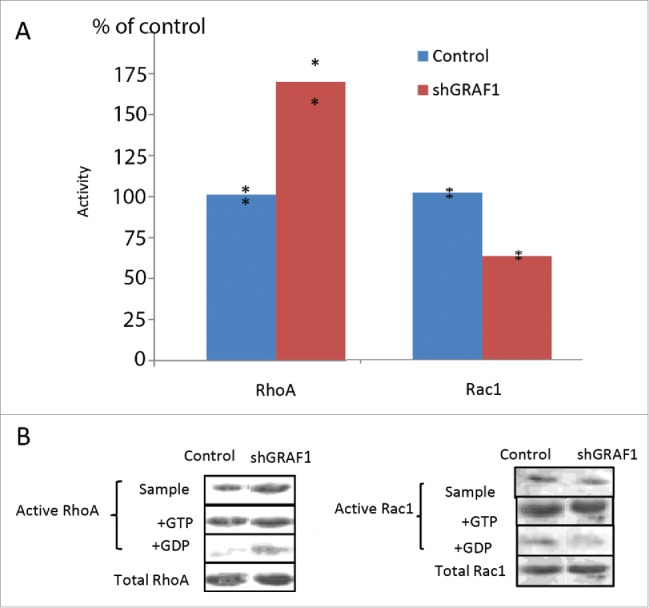
Activity of RhoA and Rac1 in control and GRAF1-depleted MCF10A cells. (A) GRAF1-depleted MCF10A cells sample showed a 75% increase in the levels of active RhoA, and a 40% decrease of active Rac1, as compared to control samples. The levels of RhoA and Rac1 activity were calculated as mentioned in Materials and Methods and expressed as percent of control. The level of the active RhoA was measured by a pull-down assay, using Rhotekin-RBD coated beads that bind the active (GTP-bound) RhoA with high affinity. Active Rac1 levels were measured by a pull-down assay, using PAK-PBD coated beads that bind the active (GTP-bound) Rac1, with high affinity. (B) The Western blot shows the level of active RhoA (left) and of active Rac1 (right) in the samples of cell lysate from control and shGRAF1-expressing cells (upper bands). GTP- and GDP-enriched cell lysates (“+GTP” and “+GDP,” respectively) were used as positive and negative controls. Amounts of pulled down, active RhoA and Rac1 were normalized relative to the total RhoA and Rac1 levels respectively, in the corresponding lysates.

To measure Rho activity, we used Rhotekin-Rho binding domain (RBD)-coated beads, which pull down only active GTP-RhoA. In a similar manner, we measured Rac activity with beads coated with the Rac binding domain of PAK-kinase ( PAK1-PBD), which pull down only active GTP-Rac1. In both cases, GTP- and GDP-enriched samples of cell lysates were used as positive and negative controls, respectively, for each RhoA and Rac1 assay. Results were normalized to total RhoA and total Rac1 levels in control and GRAF1-depleted cells and expressed as a percent of control. We thus showed that the GRAF1-depleted cells sample demonstrates a prominent increase in RhoA activity (Fig. 7A and 7B left panel). We also noticed a 40 per cent decrease in Rac1 activity in the GRAF1-depleted cells sample in comparison to the control sample (Fig. 7A and 7B right panel).

**Figure 8.**
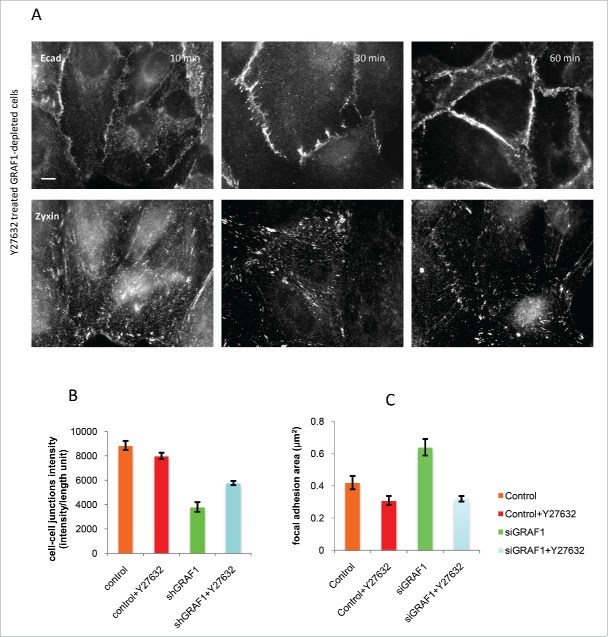
Inhibition of Rho kinase partially rescues the adhesion phenotype in GRAF1-depleted cells. (A) A gradual increase in cell-cell junction intensity (upper panel) and a decrease in focal adhesion area (lower panel) were seen during 10 to 60 min incubation of GRAF1-depleted cells with the Rho-kinase inhibitor Y27632 at a 10 µM final concentration. Immunofluorescence staining with anti-E-cadherin and anti-zyxin antibodies. Scale bar: 10 μm. (B) The average intensity of E-cadherin staining at the cell-cell junctions in the Y27632-treated GRAF1-depleted cells approached 65% of the E-cadherin intensity at the cell-cell contacts of control cells. (C) Focal adhesion area (visualized by staining with antibody to zyxin) in Y27632-treated GRAF1-depleted cells become similar to those seen in control cells treated with Y27632.

Could the increase in RhoA activity observed in GRAF1-depleted cells constitute the mechanism responsible for other phenotypic changes related to EMT, and enable identification of downstream targets of Rho that might be involved in this process? To explore this issue, we treated GRAF1-depleted cells with the Y27632 Rho-kinase inhibitor. Changes in fluorescence intensity of E-cadherin cell-cell junctions, noted at different time intervals following addition of the inhibitor, were assessed. One hour after Y27632 treatment, partial recovery of cell-cell contacts was observed, together with a decrease in average focal adhesion area, visualized by zyxin-antibody staining (Fig. 8A and C). Overall, the recovery of cell-cell junctions appeared incomplete, and the level of E-cadherin intensity never approached that of control MCF10A cells (Fig. 8B).

### Discussion

The aim of the present study was to investigate the role of the RhoGAP-domain-containing protein GRAF1 in the control of epithelial cell morphology and dynamics. The phenotypic changes we observed as a result of GRAF1-knockdown in MCF10A cells include disruption of desmosomes and cell-cell adherens junctions coupled with an E-cadherin to N-cadherin shift, decrease in epithelial markers such as cytokeratin 5 and 14 and increase in mesenchymal markers such as vimentin, snail-1 and snail-2, cell elongation and enhancement of stress fibers, increase in migration ability. These changes strongly resemble those occurring upon the EMT.^32,33^ Thus, knockdown of GRAF1 seemed to trigger the EMT in the MCF10A cells.

Previous studies showed that the EMT program and its elements, such as loss of E-cadherin and increased activity of Snail-1, highly correlate with and most probably drive the tumor malignancy.^34,35^ Consistently, we found low expression levels of the GRAF1 protein in several invasive breast cancer-derived cell lines. Moreover, the MCF7 breast cancer-derived, non-invasive cell line displayed a low GRAF1 level as well. To determine to what extent GRAF1 can restore the normal epithelial phenotype, we expressed GRAF1 in the BT-549 cell line of breast cancer origin characterized by the loss of epithelial phenotype. We obtained a significant decrease in snail-1 and snail-2 transcription factors 72 hours after expressing GRAF1 in BT-549 cell line, as compared to wild-type BT-549. A decrease in mesenchymal N-cadherin and an increase in epithelial E-cadherin were also observed. Thus, expression of GRAF1 caused changes in expression levels of the basic mesenchymal and epithelial markers consistent with the recovery of normal epithelial phenotype.

A strong indicator of neoplastic transformation in cell lines, which either lost a tumor suppressor or underwent oncogene activation, is the capacity to form anchorage-independent colonies in soft agar.^28^ Normal epithelial cells typically are unable to grow in semisolid media in the absence of the cell-matrix contact. In our experimental system, GRAF1-depleted cells formed multicellular colonies when grown in soft agar gel. These results reinforced the assumption that GRAF1 is involved in maintaining a normal phenotype.

Existing data from the literature are consistent with possible role of GRAF1 as a gene required for maintenance of normal cell phenotype. The GRAF1 gene was found to be disrupted in patients with myelodysplastic syndrome,^29^ while aberrant methylation of the GRAF1 promoter was detected in patients with acute myeloid leukemia.^30^ A recent study shows that a fusion gene between fragments of claudin 18 and GRAF1, CLDN18-ARHGAP26, found in gastric cancer, impairs epithelial integrity and induces EMT.^36^ These studies, coupled with our observations, suggest that GRAF1 indeed shares some features with tumor suppressor genes.

Possible tumor suppressor function of a protein with Rho GAP activity is not without precedent in the literature. Rho GAP known as DLC1 (“deleted in liver cancer”) is considered as a tumor and metastasis suppressor gene for several human cancers.^37^ It is demonstrated that tumor suppressor functions of DLC1 depend on its RhoGAP activity, as well as on some GAP-independent mechanisms.^38,39,40^ Following the GRAF1 knockdown in MCF10A cells, we observed a significant increase of RhoA-GTP level, suggesting that function of GRAF1 in preserving the epithelial phenotype might be mediated through its RhoGAP activity. We found also some reduction in Rac1 activity upon the GRAF1-knockdown, which may reflect a known antagonism between Rho and Rac in epithelial cells.^11^ Some features of the phenotype of GRAF1-depleted cells (enhancement of focal adhesions and stress fibers, cell elongation) can, indeed, be explained by activation of known RhoA pathway effectors, such as Rho-kinase (ROCK). It is worth noting that ROCK is also known to be associated with tumor metastases and poor clinical outcome in breast cancer.^41^ We investigated whether treatment of GRAF1-depleted cells with a ROCK inhibitor Y-27632 could rescue the normal phenotype by blocking the Rho pathway. Our results showed some recovery of the E-cadherin- positive cell-cell junctions in the Y-27632 treated GRAF1-depleted cells, but the junctional E-cadherin level was still significantly lower than that in control cells. Thus, inactivation of ROCK is insufficient for complete recovery of the normal epithelial phenotype suggesting that, besides ROCK/Rho-kinase, GRAF1 depletion activates additional effectors, related or non-related to the Rho-pathway.

In addition to RhoGAP activity, GRAF1 contains BAR and PH functional domains, which underlay its involvement in the clathrin-independent endocytosis by remodeling membranes into tubulovesicular clathrin-independent carriers (CLICs).^17^ Since GRAF1, as we showed in this study, localized to epithelial cell-cell junctions in MCF10A cells, it could in principle regulate the junctional integrity through the control of the E-cadherin endocytic trafficking. Another hypothesis concerning the GRAF1 BAR domain function is that GRAF1 may participate in a crosstalk between the Rho activity and membrane tension. It is now becoming increasingly clear that membrane tension plays an important role in the regulation of cell adhesion and migration,^42,43^ but the mechanisms via which the membrane tension affects the cytoskeletal functions remain elusive. Recent studies^44,45^ indicate that membrane tension may strongly affect recruiting BAR domain proteins to the membrane-remodeling sites in the cell. Since GRAF1 contains both BAR and RhoGAP domains, this can provide a mechanism for the membrane tension dependent local regulation of Rho functions.

Thus, future studies of GRAF1 may shed light on the basic mechanisms of the membrane-cytoskeleton crosstalk, as well as the major principles governing maintenance of the epithelial phenotype and its deterioration in the course of neoplastic transformation.

## Materials and methods

### Cell cultures and treatments

a. MCF10A cells were obtained from American Type Tissue Culture (ATCC) and cultured in DMEM/F12-(HAM)1:1 nutrient mixture (Biological Industries) supplemented with 5% horse serum (Biological Industries), insulin 0.25 IU/ml (Biological Industries), hydrocortizone 0.5μg/ml (Sigma), cholera toxin 100ng/ml (Sigma), EGF 20ng/ml (BioVision) and 1% penicillin-streptomycin solution (Biological Industries). Trypsin-EDTA 0.05% (Biological Industries) was used to subculture the cells.

Assessment of cells treated with Rho-kinase inhibitor Y27632 (Calbiochem) at a 10μM final concentration was performed at 10, 30 and 60 min., respectively.

b. MCF-7 breast cancer-derived cells were cultured according to a protocol previously described by Caramussa et al.^46^ Growth medium lacking fetal calf serum (FCS), 0.25% FCS, and 10% FCS, respectively, were used.

c. MDA-MB-231 metastatic breast adenocarcinoma cancer cells were cultured according to Brinkley et al.^47^ Growth medium lacking fetal calf serum (FCS), 0.25% FCS, and 10% FCS, respectively, were used.

d. BT-549 cells were obtained from ATCC and cultured in RPMI growth medium (Gibco) supplemented with 10% fetal bovine serum (HyClone) and 0.023 IU/ml insulin. Phosphate-buffered saline (PBS) (Biological Industries) was used to wash the cells.

### Immunostaining and fluorescence microscopy

a. Cells processed for immunostaining were fixed and permeabilized by incubation for 3 minutes in 3 % paraformaldehyde (PFA) with 0.5% Triton X-100, followed by PBS washing and incubation in 3% PFA at room temperature for an additional 20 minutes. Before staining, cells were treated with 1% BSA for 10 minutes, then immunostained by sequential incubation with the primary and secondary antibodies, for one hour each. After washing with PBS, cells were mounted in Elvanol (Mowiol 4–88, Hoechst). For microtubule staining, cells were fixed and simultaneously permeabilized at 37°C in a mixture of 3% paraformaldehyde, 0.25% Triton X-100 (Sigma), and 0.2% glutaraldehyde for 15 minutes, and then washed twice in PBS for 10 minutes each time. Before staining, cells were treated with sodium borohydride in cytoskeleton buffer (CB; containing 10 mM MES, 150 mMNaCl, 5 mM EGTA, 5 mM MgCl2 and 5 mM glucose, pH 6.1) for 15 minutes on ice. For GRAF1 staining, cells were fixed for 20 minutes in 3% paraformaldehyde at 37°C, followed by PBS washes 3 times. than incubated with antibodies for GRAF1 staining in 1% goat serum and 0.1% saponin.

b. The following primary antibodies were used for staining: mouse monoclonal anti-paxillin (1:500; BD Biosciences), mouse monoclonal anti-α -tubulin (1:500; BD Biosciences), rabbit polyclonal anti-β-catenin (1:500; Sigma-Aldrich), mouse monoclonal anti -E-cadherin (1:100; BD Biosciences), mouse monoclonal anti- cytokeratin 5 (1:100; Abcam),mouse monoclonal anti -cytokeratin 14 (1:100; Abcam), rabbit-polyclonal anti-N-cadherin (1:100; Abcam), rabbit polyclonal anti-GRAF1(1:100; Abcam). Mouse monoclonal anti -Vimentin (1:100) and rabbit polyclonal anti-Zyxin (1:200) were kindly provided by Prof. Geiger's laboratory.The same primary antibodies were used for Western blots. Secondary antibodies used: goat anti-rabbit or anti-mouse, conjugated to Cy5 (1:40; Jackson Laboratories), Alexa 488 fluorochromes (1:200; Jackson Laboratories), Cy3 goat anti-rabbit (1:200, Jackson Laboratories). F-actin was visualized using a TRITC-phalloidin probe (Sigma-Aldrich). For Western blots, secondary peroxidase-conjugated AffiniPure Goat anti-mouse and anti-rabbit antibodies (1:10,000; Jackson Laboratories) were used.

c. Images of fixed cells were recorded using the DeltaVision System (Applied Precision) that included an Olympus IX71 inverted microscope equipped with a CoolSnap HQ camera (Photometrics), a 100 W mercury lamp, and excitation and emission filter wheels. Images were acquired with an Olympus plan ApoN 60´ 1.42 N/A objective. For live-cell imaging, time-lapse recordings of control, GRAF1-depleted and GRAF1-GFP transfected cells were performed at 20-minute intervals, with a total duration of 20 hours.

d. For analysis of FA characteristics, images of paxillin-stained cells were segmented using the WaterShed algorithm, and analyzed using the Priism software package. Areas of individual FAs, FA elongation (aspect ratio) and number of FAs per cell were determined. FA characteristics were measured in at least 50 individual cells. Aspect ratio of individual cells and of cell-islands was calculated using “fit-ellipse” routine of ImageJ software. In movies, cell migration velocity was measured for 100 cells in each group, using the Track algorithm. Analysis of cell-cell contact intensity was undertaken, using the IMAGEFILE algorithm. Intensity/unit length of cell-cell contacts in 50 groups of cells from each sample was measured. Significance was assessed, using the student's T-test.

### Transfections and infections

a. The hGRAF1-EGFP plasmid was a kind gift of Harvey McMahon (MRC Laboratory of Molecular Biology, Cambridge, UK).Twenty-4 hours prior to transfection, cells were plated on glass coverslips coated with fibronectin (20 μg/ml). Transfections of MCF10A and BT-549 cell lines were performed in 36-mm dishes using X-tremeGENE HP (Roche), according to manufacturer's instructions. Six hours after transfection, cells were washed with warm growth medium without antibiotics, and incubated for 20 hours at 37 degrees in the growth medium. Immunofluorescent staining and RNA extraction were performed 48 hours after transfection, and lysate was prepared for Western blot 72 hours after transfection.

b. siRNA knockdown of GRAF1 was performed with Dharmacon SMARTpool siGENOME siRNA targeted against B-isoform of GRAF1; Dharmacon siGENOME RISC-free siRNA, a chemically modified siRNA, was used as a negative control. Validation of siRNA targeting was done by transfection of each of the 4 individual RNA sequences of the SMARTpool siGENOME:

GAAAGAAUCUAGCUUCAG;

CAUAGGAGAUGCAGAAACA;

CCAAACAGCAUCCUUAAUU;

GAACAUGACUCAGAAACUUU.

c. shRNA knockdown of GRAF1 and establishment of a stable sub-line of MCF10A cells depleted of GRAF1 were obtained by MISSION shRNA LentiviralClone infection (Sigma), containing shRNA directed against the GAP domain of GRAF1. Infected cells were selected using puromycin treatment.

### Real-time PCR

RNA extraction was performed with an RNAeasy MicroKit (Qiagen), according to manufacturer's instructions. qPCR was carried out using DyNAmoTM Flash SYBR Green qPCR Kit (Finnzymes) and the Stratagene Mx3000P QPCR System. We used the following primers:

for GRAF1:

5′-CCGTCAGGTCTGTTGCAGGGT-3′; 5′-CGGAACGGTGTGCTGGAGTCT 3′;

for β-actin:

5′-GTACCACTGGCATCGTGATGGACT-3′;

5′-CCGCTCATTGCCAATGGTGAT-3′.

### Anchorage-independent growth assay

In each well of a 6-well dish, MCF10A or shGRAF1 MCF10A cells were seeded in 0.6% agar, and covered with 0.3% agar. Agar layers contained DMEM supplemented with 5% horse serum. The cells were fed with 2 ml of growth medium every 7 days, and incubated for a total of 21 d. Using an inverted Zeiss, Axioplan microscope, pictures were taken of 2 wells for each type of cell. The experiment was repeated twice.

### RhoA and Rac1 activation assays

RhoA and Rac1 activation assays were performed according to the manufacturer's instructions (Cytoskeleton). For the measurement of the activity of small G-proteins (Rho and Rac) in the control and Graf1-knockdown cells, the lysates of each type of the cells were divided into 3 samples, one of which was saturated by GTP (positive control, corresponding to the maximal activity of the G-protein in the sample), another with GDP (negative control, corresponding to the minimal, background activity of the G-protein in the sample), and the third remained non-treated. The amounts of Rho-GTP (or Rac-GTP) in these 3 samples normalized per the total levels of the Rho (or Rac) in each sample were denoted as M (maximum), B (background), and N (non-treated), respectively. The normalized G-protein activities in each type of lysate were then calculated, according to the formula A = (N-B)/(M-B).

### Scanning Electron Microscopy (SEM) imaging

Samples were fixed with 2.5% glutaraldehyde and 2.5% paraformaldehyde in 0.1M Cacodylate buffer for 1 hour, followed by OsO4 1% fixation for 1 hour, dehydrated in increasing concentrations of etanol, and dried in a BAL-TEC CPD 030 critical point drier. Samples were placed on carbon tape and sputter-coated with gold-palladium (Edwards, S150), then examined using a high-resolution Ultra 55 (Zeiss) field emission scanning electron microscope.

### Transmission electron microscopy of cell cultures

Cells were seeded on cover slips 12 mm and then fixed for 2 hours in Karnovsky-fixative (2% gluteraldehyde, 3% paraformaldehyde, 3% sucrose) in 0.1M cacodylate-buffer at room temperature, washed 4 times in 0.1M cacodylate-buffer and kept at 4°C until further processing. Cells were then post-fixed with 1% OsO4, 0.5% K_2_Cr_2_O_7_, 0.5% K_4_[Fe(CN)_6_].3H_2_O, 3% Sucrose in 0.1M cacodylate-buffer for 2 hours at room temperature, and washed twice with 0.1M cacodylate-buffer, 3% Sucrose and 3 times in double distilled water (DDW). Cells were then stained with 2% uranyl-acetate for 1 hour, washed with DDW and dehydrated in Ethanol and embedded inEpon EMBED 812(EMS). TheEpon-blocks were then roughly cut into small pieces, aligned, and re-embedded. Frontal sections, 70–100 nm were cut with Leicaultracat UCT and analyzed in a C-12 Spirit FEI electron microscope; pictures were taken with an Eagle CCD-camera 2kx2k.

## Supplementary Material

Supplementary_materials.zip
