# Supplementary material for: Involvement of Rho GAP GRAF1 in maintenance of epithelial phenotype
Source: Cell Adh Migr. 2016 Sep 2;11(4):367–83. doi: 10.1080/19336918.2016.1227910 (PMC5569970; doi:10.1080/19336918.2016.1227910)
Supplement: Supplementary_materials.zip [file kcam-11-04-1227910-s001.zip › Supplementary Table 1 and Legends.docx]

**Supplementary Figure 1: Additional information concerning GRAF1 knockdown cells.**

**A.** The microtubule network visualized by immunofluorescence staining with anti-alpha-tubulin antibody is not affected by GRAF1 knockdown. This justifies using alpha -tubulin as a loading control in our experiments. Scale bar: 10 μm**. B.** Western blot analysis of the effects of individual siRNAs from “SMARTpool ” on GRAF1 expression levels. **C.** Quantification of the Western blot shown in **B.**

**D-H.** Effect of individual “SMARTpool ” siRNAs on the cell-cell adherens junctions, as visualized by immunofluorescence staining with anti-E-cadherin (left column) and anti-beta-catenin antibodies (right column), respectively. **D.** Control MCF10A cells. **E-H.** Cells transfected with si1, si2, si3 and si4, respectively. Scale bar: 10 μm.

**Video 1**: Migration pattern of MCF10A control cells

**Video 2**: Migration pattern of GRAF1- depleted MCF10A control cells

**Supplementary Table 1. Characteristics of the breast cancer-derived cell lines in which GRAF1 was found to be expressed at low levels**. Most of the breast cancer cell lines are invasive, with epithelial or polymorphic phenotypes.

| **Invasiveness** | **Morphology in culture** | **Tissue origin** | **Cell line** |
| --- | --- | --- | --- |
| A slow-growing cell line  *Soule HD, et al., J. Natl. Cancer Inst, 1973* | Epithelial | Mammary gland/breast; derived from metastatic site: pleural effusion | MCF7 |
| High  *Satram-Maharaj T. et al., Cell Signal., 2014* | Epithelial | Mammary gland/breast; derived from metastatic site: pleural effusion | MDA-MB-231 |
| High  *Checkun S. et al., Exp Oncol., 2013* | Epithelial | Mammary gland; breast: pleural effusion | MDA-468 |
| High  *Gordon LA et al., Int. J. Cancer, 2003* | Polymorphic | Mammary gland/breast; derived from metastatic site: pleural effusion | MDA-436 |
| Density-dependent (high in low density)  *Nerlich AG. et al., Oncol Lett,2013* | Spindle shaped | Mammary gland/breast; derived from metastatic site: pleural effusion | MDA-435 |
| High  *Hoffmeyer MR et al., Cancer Cell International 2005* | Epithelial | Primary Inflammatory breast cancer | SUM-149 |
