# Supplementary figures and images for: Involvement of Rho GAP GRAF1 in maintenance of epithelial phenotype
Source: Cell Adh Migr. 2016 Sep 2;11(4):367–83. doi: 10.1080/19336918.2016.1227910 (PMC5569970; doi:10.1080/19336918.2016.1227910)

Supplementary Fig.1

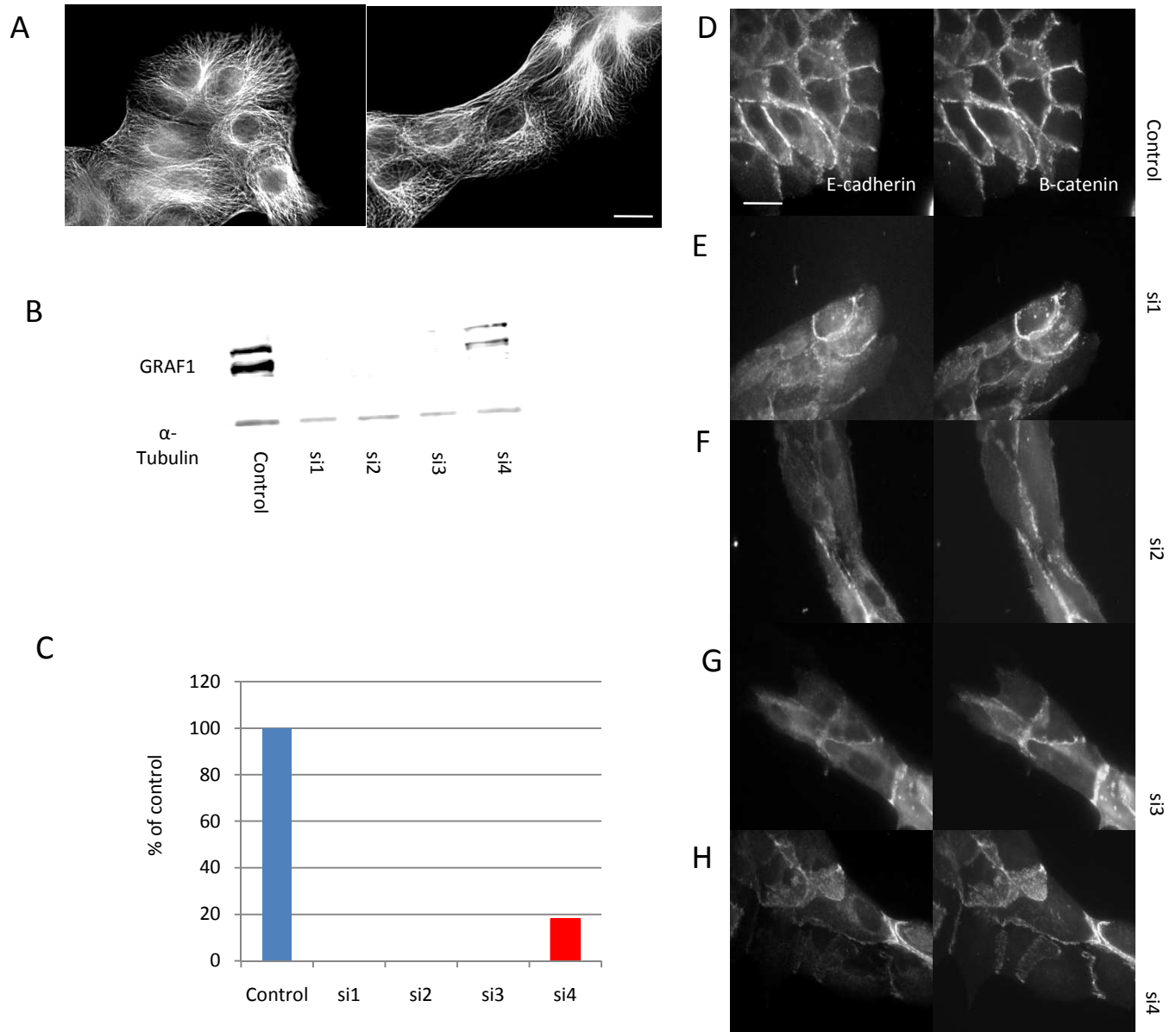

Supplement: Supplementary_materials.zip [file kcam-11-04-1227910-s001.zip › Supplementary Fig 1.pdf]
